# Supplementary material for: Reduced SLIT2 is Associated with Increased Cell Proliferation and Arsenic Trioxide Resistance in Acute Promyelocytic Leukemia
Source: Cancers (Basel). 2020 Oct 27;12(11):3134. doi: 10.3390/cancers12113134 (PMC7693375; doi:10.3390/cancers12113134)
Supplement: Supplementary file 1 [file cancers-12-03134-s001.zip › cancers-965365-supply/Cancers-965365-supplementary.pdf]

Supplementary Materials

Reduced SLIT2 is Associated with Increased Cell Proliferation and Arsenic Trioxide Resistance in Acute Promyelocytic Leukemia

Isabel Weinhäuser, Diego A. Pereira-Martins, Cesar Ortiz, Douglas R. Silveira, Luíse A. A. Simões, Thiago M. Bianco, Cleide L. Araujo, Luisa C. Koury, Raul A. M. Melo, Rosane I. Bittencourt, Katia Pagnano, Ricardo Pasquini, Elenaide C. Nunes, Evandro M. Fagundes, Ana B. Gloria, Fábio Kerbauy, Maria de Lourdes Chauffaille, Armand Keating, Martin S. Tallman, Raul C. Ribeiro, Richard Dillon, Arnold Ganser, Bob Löwenberg, Peter Valk, Francesco Lo-Coco, Miguel A. Sanz, Nancy Berliner, Emanuele Ammatuna, Antonio R. Lucena-Araujo, Jan Jacob Schuringa and Eduardo M. Rego

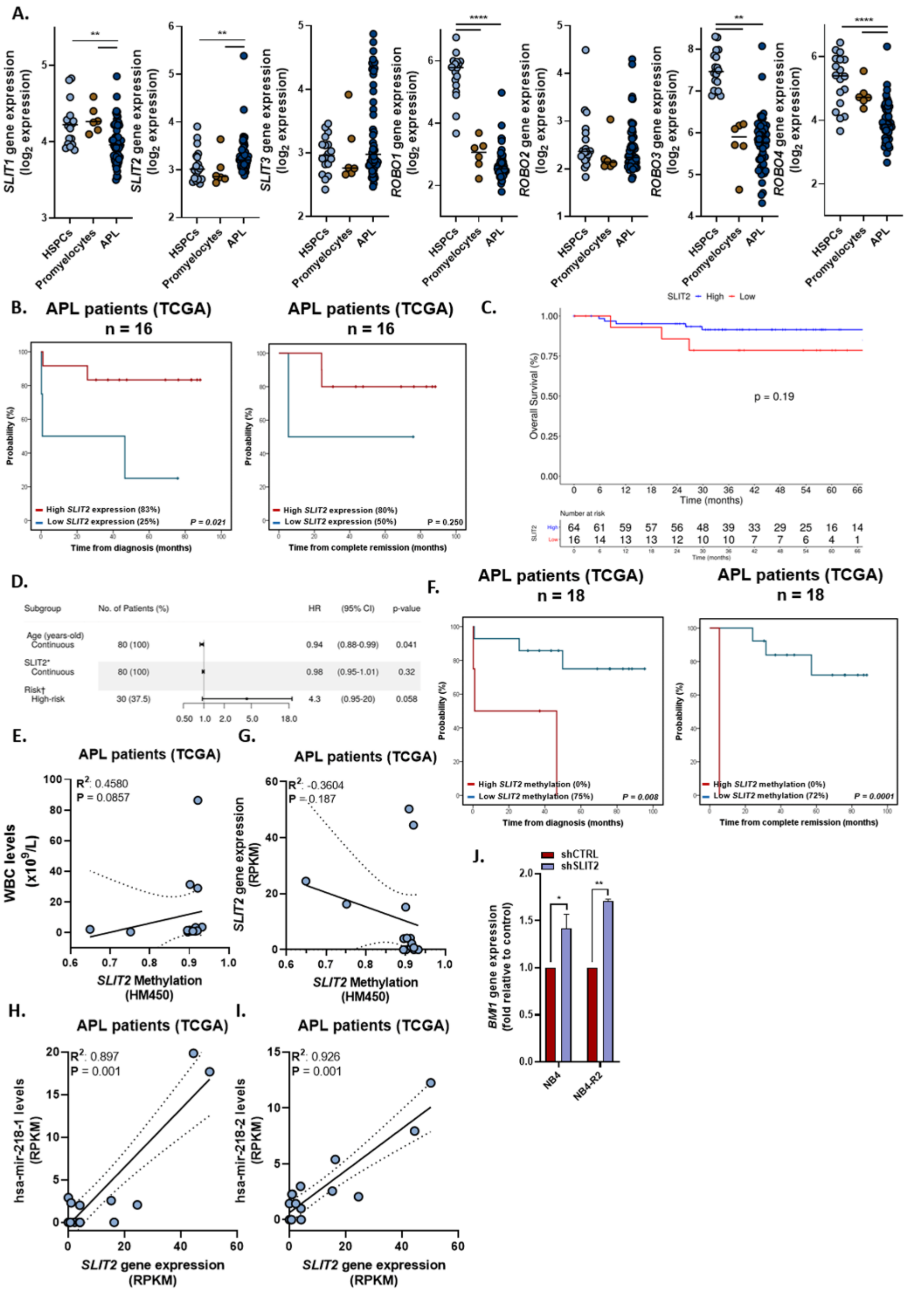

Figure S1. SLIT-ROBO expression in APL patients (**A**) SLIT-ROBO transcript levels were evaluated in acute promyelocytic leukemia patients (APL) compared to healthy hematopoietic stem- and progenitors-cells (HSPCs) and healthy promyelocytes using the BloodSpot dataset. Gene expression is expressed as log2 values. Horizontal lines represent the median. $*p < 0.05$, $** p < 0.01$, $****p < 0.0001$. (**B**) The probability of overall survival and disease-free survival in APL patients from the TCGA study according to the SLIT2 expression. Survival curves were estimated using the Kaplan-Meier method and the log-rank test was used for comparison. (**C**) The probability of disease-free survival in APL patients from the ICAPL consortium (**D**) Multivariate Cox model for overall survival. (**E**) Correlation analysis of SLIT2 global methylation and white blood cell count in APL patients included in the TCGA study. (**F**) The probability of overall survival and disease-free survival in APL patients from the TCGA study according to the SLIT2 global methylation levels. Survival curves were estimated using the Kaplan-Meier method and the log-rank test was used for comparison. (**G**) Correlation analysis of SLIT2 global methylation and SLIT2 gene expression in APL patients included in the TCGA study. Correlation analysis of SLIT2 gene expression and the expression of hsa-mir-218-1 (**H**) and hsa-mir-218-2 (**I**) in APL patients included in the TCGA study. (**J**) Quantitative gene expressions of BMI-1 were analyzed in shSLIT2 cells relative to the shScrambled cells. Values were normalized to the expression level of housekeeping genes (ACTB and RPL30). Results are shown as mean ± standard error mean (SEM) of, at least, 4 independent experiments. Each independent experiment was performed in triplicate. Student's *t*-test was used for statistical analysis.

© 2020 by the authors. Licensee MDPI, Basel, Switzerland. This article is an open access article distributed under the terms and conditions of the Creative Commons Attribution (CC BY) license (http://creativecommons.org/licenses/by/4.0/).
